# Marginal value analysis reveals shifting importance of migration habitat for waterfowl under a changing climate

**DOI:** 10.1002/ece3.10632

**Published:** 2023-11-09

**Authors:** Ryan C. Burner, Benjamin D. Golas, Kevin J. Aagaard, Eric V. Lonsdorf, Wayne E. Thogmartin

**Affiliations:** ^1^ U.S. Geological Survey Upper Midwest Environmental Sciences Center La Crosse Wisconsin USA; ^2^ Department of Biology Colorado State University Fort Collins Colorado USA; ^3^ AE Strategies McLean Virginia USA; ^4^ Department of Environmental Sciences Emory University Atlanta Georgia USA

**Keywords:** *Anas platyrhynchos*, dabbling duck, energetics, marginal value analysis, migration, National Wildlife Refuges

## Abstract

Migratory waterfowl are an important resource for consumptive and non‐consumptive users alike and provide tremendous economic value in North America. These birds rely on a complex matrix of public and private land for forage and roosting during migration and wintering periods, and substantial conservation effort focuses on increasing the amount and quality of target habitat. Yet, the value of habitat is a function not only of a site's resources but also of its geographic position and weather. To quantify this value, we used a continental‐scale energetics‐based model of daily dabbling duck movement to assess the marginal value of lands across the contiguous United States during the non‐breeding period (September to May). We examined effects of eliminating each habitat node (32 × 32 km) in both a particularly cold and a particularly warm winter, asking which nodes had the largest effect on survival. The marginal value of habitat nodes for migrating dabbling ducks was a function of forage and roosting habitat but, more importantly, of geography (especially latitude and region). Irrespective of weather, nodes in the Southeast, central East Coast, and California made the largest positive contributions to survival. Conversely, nodes in the Midwest, Northeast, Florida, and the Pacific Northwest had consistent negative effects. Effects (positive and negative) of more northerly nodes occurred in late fall or early spring when climate was often severe and was most variable. Importance and effects of many nodes varied considerably between a cold and a warm winter. Much of the Midwest and central Great Plains benefited duck survival in a warm winter, and projected future warming may improve the value of lands in these regions, including many National Wildlife Refuges, for migrating dabbling ducks. Our results highlight the geographic variability in habitat value, as well as shifts that may occur in these values due to climate change.

## Graphical abstract

To quantify the marginal value of waterfowl habitat across the contiguous United States, we used a continental‐scale energetics‐based model of daily dabbling duck movement during the non‐breeding period in both a warm and a cold winter. We quantified the effect of removing each habitat grid cell on overall duck survival. We found that the importance of habitat depended not only on the resources it contained but also on location (especially latitude) and local weather patterns.

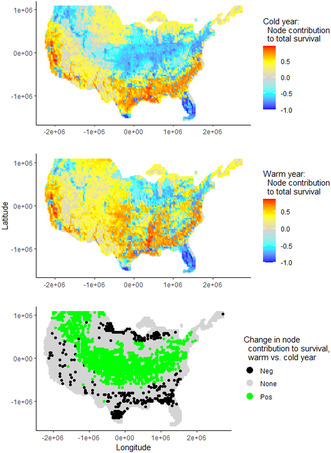

## 1 INTRODUCTION

Migratory waterfowl are the focus of much consumptive and non‐consumptive use in North America, supplying substantive economic and cultural benefits (Mattsson et al., 2018; U.S. Fish and Wildlife Service, 2018). As such, they have long been the focus of conservation efforts on public and private lands and have been studied extensively (Brasher et al., 2019). Habitat availability and climate in the breeding season are important for waterfowl productivity, but high‐quality migration and wintering habitat are also imperative for maintenance of populations (Newton, 2006). For example, spring body condition, which reflects migration forage and winter weather, predicts reproductive success in mallard (Devries et al., 2008; Osnas et al., 2016).

Migration and winter habitat for waterfowl consists of a complex matrix of private and public lands. For dabbling ducks in particular, waste grain in agricultural fields is an important food source (Pearse et al., 2012; Stafford et al., 2006), complemented by more aquatic habitat (Hagy et al., 2014; Herbert et al., 2021). The National Wildlife Refuge (NWR) system, managed by the U.S. Fish and Wildlife Service (USFWS), plays an important role across the migration and wintering ranges of these species and includes over 400 properties set aside for conservation in the contiguous United States (Hamilton et al., 2015). Refuges play a central role in protecting high‐quality waterfowl habitat, and the USFWS coordinates these management efforts nationally in part through the Integrated Waterfowl Management and Monitoring program (Aagaard et al., 2017). The value of a given tract of habitat, however, is determined not only by the resources that it provides but also by its geographic location and the continental‐scale weather patterns in a given year (Lovvorn, 1989; Schummer et al., 2017). Migrating waterfowl face a host of decisions, from the timing and route of migration to the distance traveled, leading to complex trade‐offs between distance to breeding ground and likelihood of encountering severe weather (Aagaard et al., 2022), particularly in early spring and late fall (Si et al., 2015). These trade‐offs mean that habitat at different latitudes likely varies in importance (Lonsdorf et al., 2016), and these patterns vary among flyways and years with differing weather patterns (Meehan et al., 2021; Schummer et al., 2017).

Most of the contiguous United States is projected to become much warmer in the coming decades, particularly in winter (Deser et al., 2012). Precipitation projections have higher uncertainty and more spatial variability, but some regions will likely become drier in winter (West and Southeast), whereas others (Great Lakes, Northern Great Plains) may experience an increase in winter precipitation (Deser et al., 2012). The spring thaw, also important for bird migration, is predicted to occur earlier (Rawlins et al., 2016), potentially allowing waterfowl to move northward more rapidly (Lehikoinen et al., 2019) where they will face a higher risk of extreme weather events in early spring. These persistent directional changes in climate in coming decades (Deser et al., 2012; Rawlins et al., 2016) may change the routes and phenology of migratory waterfowl in North America (Aagaard et al., 2018, 2022; Notaro et al., 2016), thereby changing the relative importance of local habitat based on their geographic distribution. Protected areas like the NWR system and conservation initiatives such as the Conservation Reserve Program, which were not designed to anticipate future climatic shifts, may have gaps in coverage and suboptimal geographic allocations of resources. It is therefore important to understand which lands (in which regions) are most important under current and future conditions to ensure that public and private lands continue to meet the needs of migratory waterfowl.

We used a continental‐scale energetics‐based model of daily dabbling duck movement from Aagaard et al. (2022), parameterized largely using information from mallards (*Anas platyrhynchos*), to assess the marginal value of lands across the contiguous United States during the non‐breeding period (September to May). We define an area's marginal value as the impact of removing that area on total continent‐wide duck survival (and duck use days (DUD)). We examined the effect of eliminating units of habitat (nodes 32 km × 32 km) in a particularly cold and a particularly warm winter, asking:

- Which habitat nodes are most important?
  - How does this vary among years with different weather patterns?
  - How does this vary within a year?
  - Are resources or geography better predictors of node importance?
- What is the relative importance of nodes containing the NWR system?
  - Are some subregions over‐ or under‐)represented in the NWR system relative to their importance?

## 2 MATERIALS AND METHODS

### 2.1 Study area

We assessed the marginal value of all lands in the contiguous United States south of the main breeding concentration of dabbling ducks during the non‐breeding period (considered to be September to May for our purposes). This includes 420 NWRs and other USFWS management units (e.g., elk or deer refuges, fish and wildlife refuges; Appendix 1).

### 2.2 Energetics‐based movement model

The continental‐scale energetics‐based mechanistic model from Aagaard et al. (2022) simulates duck movements, stopovers, and mortality through daily time steps as a function of body condition, energetics, forage and roost habitat availability, and weather conditions, parameterized largely from existing literature on mallards (*Anas platyrhynchos*). It is a deterministic model describing the relative proportion of the continental duck population expected to occur at a given node each day. Briefly, individual birds are distributed across the breeding range at the start of the simulation based on NatureServe range maps (Ridgley et al., 2005) and breeding population survey data; refer to Lonsdorf et al. (2016). For simplicity, all ‘migration’ nodes are considered free of ducks on day zero. Days are simulated iteratively, with birds going through a sequential series of simulated processes each day:

*Foraging → Body mass loss/gain → Departure → Arrival → Mortality → Foraging… (repeat)*

Birds of varying body condition consume calories during stopovers to improve their condition and expend them during migrational movements to new stopover locations. Decisions to remain at a stopover to continue to improve body condition or to depart the stopover to continue migration are a function of present body condition (measured as grams of fat available for flight), daily net change in body condition (due to foraging gains and metabolic loss), weather severity index (WSI; described below), and distance to the closest breeding node (Aagaard et al., 2022; Lonsdorf et al., 2016). The poorer the habitat and weather conditions faced by birds at a stopover, the greater their probability of departure for more suitable locations (Aagaard et al., 2022; Lonsdorf et al., 2016; O'Neal et al., 2018). This model is applied to the non‐breeding period to evaluate movement patterns in the face of historical weather conditions (weather data used in this study are described below).

In the model, the migratory zone of the continental study area is divided into 6950 grid cells (32 × 32 km), which we refer to as nodes (Appendix 2). All habitat and weather covariates (described below) are summarized at the scale of these nodes. We use Albers equal area conical projection, which ensures that each node is very nearly the same size regardless of latitude (Snyder, 1982). The probability of departing a given node on a given day is a function of bird body condition, local weather, and forage availability. Departure probability increases with increased body condition, reduced forage availability, and increased WSI as described by equations in Aagaard et al. (2022). Upon departure, birds are distributed among other nodes based on node distance from breeding ground, roosting and foraging habitat availability, and weather. Weather is important for determining the timing and routes of waterfowl migration, as demonstrated by empirical work (Masto et al., 2022; Weller et al., 2022) and mechanistic modeling (Aagaard et al., 2022; Lonsdorf et al., 2016). Probability of mortality is a function of body condition, with poorest condition birds facing greatest risk, following the equations in Aagaard et al. (2022). The model is parameterized based on a large body of empirical work on mallards, dabbling ducks, and other waterbirds (Aagaard et al., 2022).

Daily historical weather data (temperature, frozen precipitation, and air density) were extracted from the National Oceanic and Atmospheric Association's National Centers for Environmental Prediction National Center for Atmospheric Research Reanalysis Project (NOAA NCEP) based on Kalnay et al. (1996). Temperature and snowfall were combined to produce the WSI of Schummer et al. (2010) as described in appendix 3 of Aagaard et al. (2022). Calories of forage available in each node (Appendix 2) were estimated by Lonsdorf et al. (2016) based on the National Land Cover Database (Fry et al., 2011) and Center for Topographic Information (2009), as was amount of roosting habitat. For these caloric values, justifications, and references, refer to appendices S1 & S3 in Lonsdorf et al. (2016). Lonsdorf et al. (2016) estimated shoreline as the sum of all 30‐m pixels of open water bordering land. An expanded future version of this model is planned to include duck mortality due to hunter harvest, but this amendment was beyond the scope of the current analyses.

### 2.3 Node knockouts for marginal value analysis

The marginal value of a location is the relative value (in duck survival and DUD) added or lost to the system by the presence of that location. To determine the marginal value of each migration node (Appendix 2), we first ran the migration model through an entire non‐breeding period (273 days, from September 01 to May 31) to establish a baseline for each modeled year (described below). We then proceeded to ‘knockout’ each of these migration nodes (*n* = 6950), one at a time, and run the model through the full non‐breeding period again. We followed Lonsdorf et al. (2016) and Aagaard et al. (2022) in starting 19,856,514 dabbling ducks distributed across their core breeding range on day zero (Lonsdorf et al., 2016). We defined migration nodes as nodes outside this core breeding range and below 32.6° north latitude. To knock out a node, we set forage and roosting habitat in that node each equal to zero, which reduced the probability of birds arriving at that node to zero. No birds started in our non‐breeding nodes, so knockouts did not affect total number of birds at time zero. Taking the difference in a given metric (described below) between each simulation and the baseline scenario (all nodes present) quantified the marginal value of each node.

We ran the full 273‐day simulation independently for each node knockout, using consistent starting conditions. To determine the effects of weather on node marginal value, we repeated this simulation using climate data from each of 2 years. We selected 1956–1957 (hereafter referred to as 1957) as a particularly cold winter (Ludlum, 1957) and 2014–2015 (hereafter 2015) as a particularly warm winter (Aagaard et al., 2022; Appendix 3). Precipitation levels also differed among the years. To determine how marginal value varied with weather, we ran each of the node knockout simulations separately using climate data for each of these years and compared each to a baseline run of the model that included all nodes in the appropriate year. To save time, simulations for multiple nodes were run in parallel using the *doParallel* R‐package (Microsoft Corporation and Weston, 2020). All analyses were completed in R (R Core Team, 2022). Computation time constraints prevented us from running the marginal value analysis across all project years, so we restricted our analysis to comparing extremes (a warm vs. cold year).

The effects of removing a node, that is, its marginal value, were assessed using two metrics: change in total number of surviving ducks (SURV) at the end of the simulation period, and change (Δ) in total DUD over the full continent and non‐breeding period. Total survival is the sum number of living ducks on the final day (Day 273; $\sum_{j=1}^{6950}N$). Total DUD is the cumulative sum of all living ducks (*N*) occurring in each node across all 273 simulated days ($\sum_{d=1}^{273}\sum_{j=1}^{6950}N$), where *j* = 1–6950 nodes and *d* = 1–273 days. We express the marginal value of nodes in terms of Δ DUD (DUD_baseline_ − DUD_knockout_) and Δ SURV (SURV_baseline_ − SURV_knockout_), such that a positive value represents a net positive contribution (i.e., marginal value) of a node and a negative value a negative contribution. SURV is important because of its effects on population dynamics and DUD is a useful metric because it is commonly used by waterfowl managers to quantify total duck use of an area through time (Krainyk et al., 2021).

The effect of a node on SURV can differ in magnitude (and, rarely, in direction) from its effect on DUD. For example, excess mortality (i.e., additional total mortality relative to the baseline scenario) resulting from a node knockout will cause a much higher reduction in total DUD (which are summed across all simulation days) if that mortality occurs early (vs. late) in the non‐breeding period. To quantify this effect, we recorded the day on which the median excess mortality occurred (MORTDAY) for each node knockout. To do this, we calculated the first day on which daily total duck numbers in each node knockout simulation were lower than the baseline total duck numbers by a margin of Δ SURV/2 for that node and year. Some node knockouts, through removal of deleterious habitat, increased SURV and DUD; in these cases, no value was calculated for MORTDAY, but we instead estimated the median date on which excess survival (SURVDAY) occurred using a similar calculation.

For display and analysis purposes, we log‐transformed Δ DUD and Δ SURV to reduce the influence of extreme values. Positive and negative values were log‐transformed separately; negative values (node contributed negatively) were first multiplied by −1, then log‐transformed and again multiplied by −1 to restore the original sign. Positive values (node contributed positively) were simply log‐transformed. All transformed values (positive and negative) were then scaled proportionately such that the maximum absolute value of any positive or negative value was 1. This arrangement allows direct comparison of positive and negative values because they are scaled identically.

### 2.4 Contributions of each NWR

To assess the marginal value of the NWRs in the contiguous United States for migrating dabbling ducks, we used the ‘FWS National Realty Tracts’ shapefile of all tracts of NWR land from the USFWS (https://gis‐fws.opendata.arcgis.com/) to determine the node into which each portion of each refuge fell. We did this using the ‘join attributes by nearest’ tool in QGIS (qgis.org) and included all 420 refuges (62,800 km^2^) and other USFWS units (hereafter ‘refuges’) occurring within our study area (Appendix 1).

Many refuges had tracts in more than one node (Appendix 1), so to summarize the marginal value of the nodes containing each refuge, we used a weighted average of the fields of interest (Δ DUD and Δ SURV), averaged across the nodes in which each tract fell, weighted by the total area of tracts for that refuge in that node. Our mean values by refuge thus represent the weighted mean marginal value of the node(s) in which that refuge is located. Of course, not all refuges were designed with waterfowl in mind, but we nonetheless include all refuges to give an overview of the refuge system as a whole (for marginal value of nodes containing individual refuges, refer to Appendix 1).

## 3 RESULTS

### 3.1 Relative node marginal values

**FIGURE 1:**
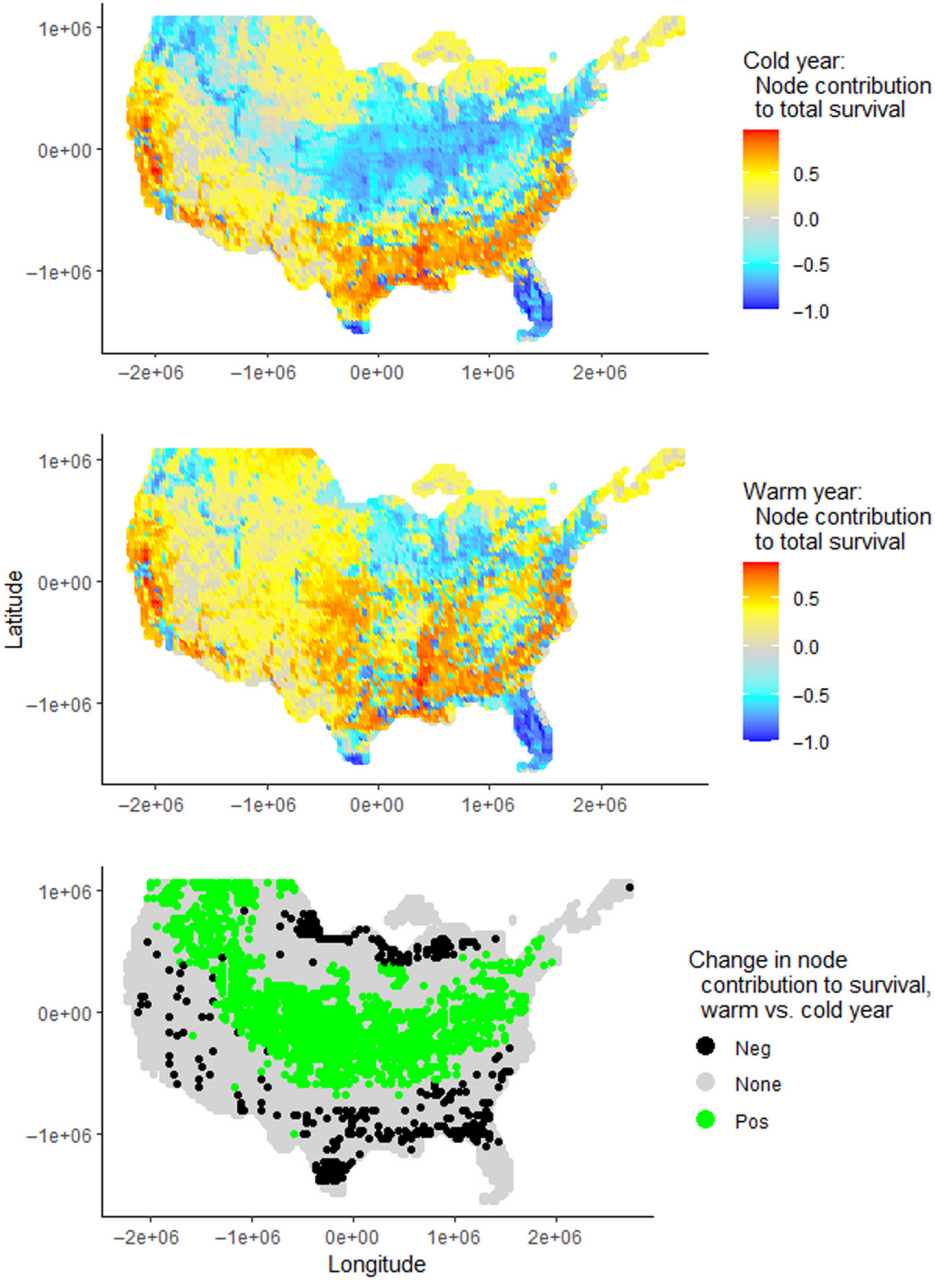
Relative contributions (i.e., marginal value) of migration nodes to total survival of dabbling ducks throughout the non‐breeding period in a relatively cold (top; 1957) and a relatively warm (center; 2015) year. The bottom panel shows nodes that became positive (pos) and negative (neg) in a warm winter, relative to a cold winter. All estimates are based on single‐node knockouts in an energetics‐based movement and foraging model (Aagaard et al., 2022). To better display the variation among nodes while reducing the influence of extreme values, positive values were log‐transformed. For negative values, the absolute value was log‐transformed and the negative sign was then restored. Positive and negative values were each then scaled proportionately to each other for easy comparison. All maps use Albers equal area conical projection centered on the contiguous United States.

Node knockouts revealed that nodes varied widely in their contributions to SURV (i.e., marginal value; Figure 1) and total DUD (Appendix 4). Patterns in total DUD were similar to SURV, so we relegated those findings to Appendix 4 (also refer to Appendix 1) and focus most inferences on SURV. Generally, irrespective of weather year, nodes in the Southeast, central East Coast, and California made the largest positive contributions to SURV. Conversely, nodes in the Midwest, Northeast, Florida, and Pacific Northwest negatively impacted survival, such that SURV increased when these nodes were removed. Much of the West had lower marginal value. Nevertheless, some nodes were consistent contributors or detriments, irrespective of weather year. For instance, the Mississippi Alluvial Valley and California Central Valley contributed generally equally well between years, as might be expected given their prominent role in providing wintering habitat for dabbling ducks, whereas the Rio Grande Valley of Texas, Florida, south of the panhandle, the southern terminus of the Appalachians, Puget Sound, and San Francisco Bay were detrimental in both years. When comparing a cold and a warm year (Figure 1), notable differences appeared. The lower Midwest and central Great Plains, for example, had more nodes with a positive marginal value in a warm winter (Figure 1). But in a warm winter, some nodes along the Gulf Coast and Great Lakes had negative marginal value (compared with positive value in a colder winter; Figure 1).

### 3.2 Impacts of node removal on fall, winter, and spring populations

**FIGURE 2:**
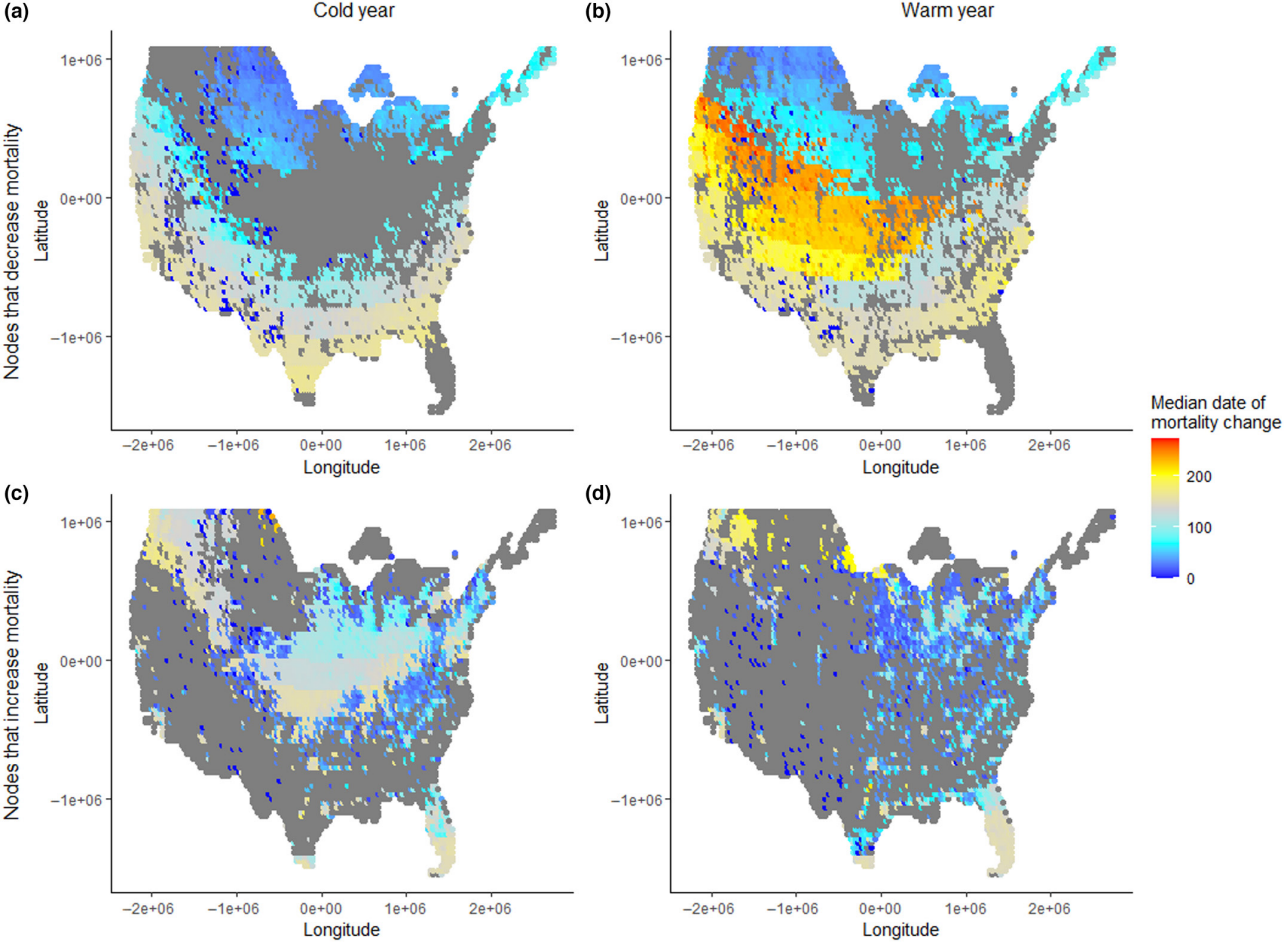
Presence of a given node can decrease (top; a, b) or increase (bottom; c, d) total dabbling duck mortality. Plots show the day of the non‐breeding season on which the median excess mortality occurs (top; MORTDAY) or is avoided (bottom; SURVDAY) when each node is removed. Estimates are shown for a cold (left; a, c) and a warm winter (right; b, d). Days 0, 100, and 200 correspond to September 01, December 10, and February 20, respectively. All estimates are based on single‐node knockouts in an energetics‐based movement and foraging model (Aagaard et al., 2022). All maps use Albers equal area conical projection centered on the contiguous United States.

In addition to varying in their marginal value for dabbling duck survival, nodes also varied in the time of year in which their benefits (or detriments) to duck survival manifested themselves (Figure 2). The time of year in which a node's contribution (positive or negative) to duck survival was most important can be understood by comparing duck mortality throughout the year in the presence and absence of a given node. In a cold winter, duck use of more northerly nodes that had positive marginal value resulted in decreased duck mortality (i.e., increased survival) early in the fall (Figure 2a). Nodes with positive marginal value located farther south, however, reduced mortality later in the winter. Among nodes with negative marginal value in a cold winter (Figure 2c), the season during which a given node increased duck mortality depended on its geographic location. Many nodes in portions of the Northwest, Midwest, and Great Plains increased mortality in late winter, although some nodes on the fringes of these regions appear rather to be associated with mortality early in the fall. In a warm winter, however, a large swath of the central Great Plains and central West contributed to reduced duck mortality during the spring migration (Figure 2b), and the negative effects of the Midwest and central Great Plains during late winter (Figure 2d) were somewhat reduced.

### 3.3 Predictors of node importance

**FIGURE 3:**
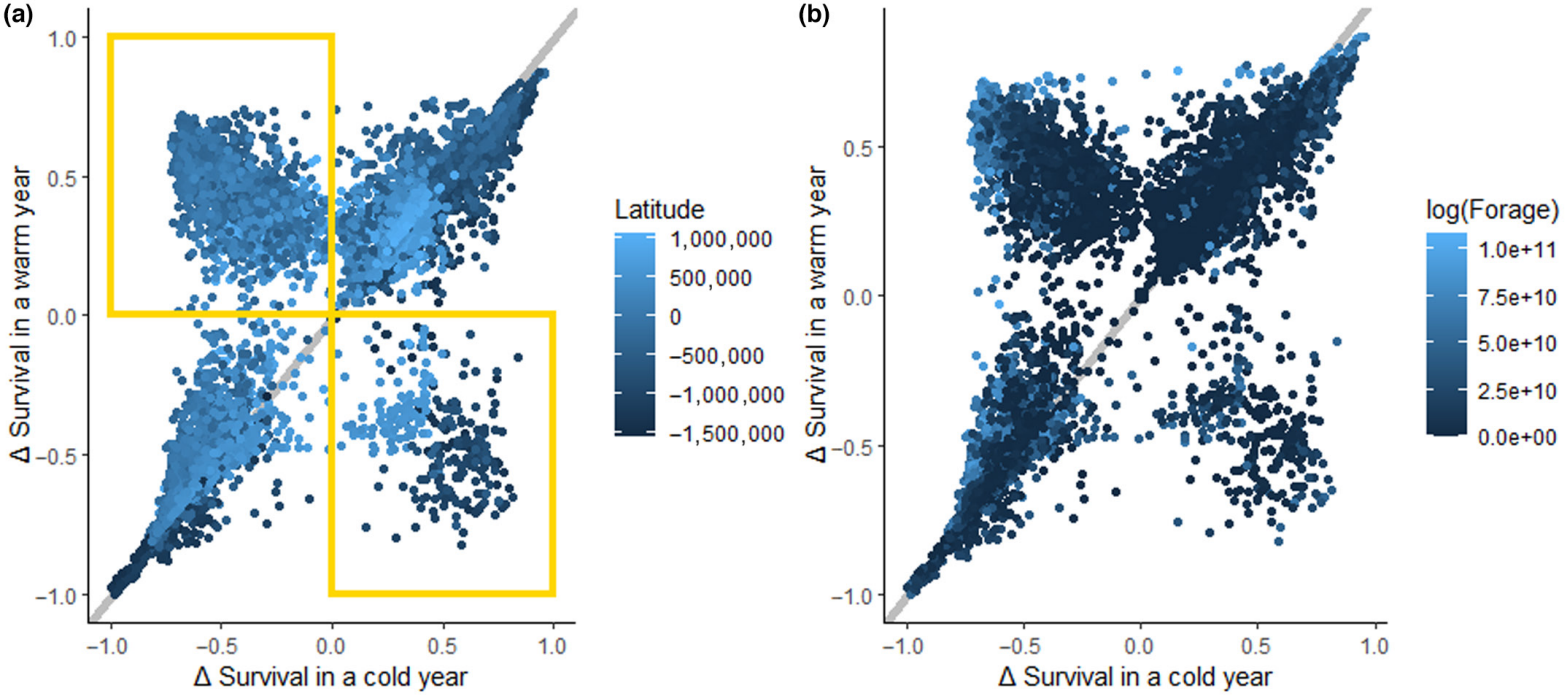
Difference in node marginal value to dabbling duck survival in a warm (*y*‐axis) versus a cold (*x*‐axis) year. Each point represents a node, colored by latitude (left; a) or forage availability (right; b). Many nodes switch to make positive contributions in a warm winter (Quadrant II; upper left), but contributions of some nodes become negative a warm winter (Quadrant IV; lower right). Marginal values of nodes at moderately high latitudes are much higher in a warm winter (left), and nodes with very high forage availability are among the nodes with higher contributions in a cold winter (right). Marginal value for survival was determined based on effects of node removal in the energetics‐based movement model of Aagaard et al. (2022). For a similar plot colored by baseline node DUD, refer to Appendix 6. Latitude is based on Albers equal area conical projection centered on the contiguous United States.

Nodes with more forage varied more widely in their marginal value for duck survival than did nodes with less forage (Appendix 5). Contrary to expectations, nodes with abundant forage often negatively affected the number of ducks surviving to the spring. Approximately a quarter of nodes differed between warm and cold winters in their contribution to the number of ducks surviving to spring, largely as a function of node latitude and forage availability (Figure 3), which affected baseline DUD (Appendix 6). A large subset of nodes at moderately high latitudes (e.g., the latitude of the Midwest) had a negative impact on survival in a cold winter but had a large positive impact in a warm winter (Figure 3a, upper left quadrant, 20.6% of all nodes), and the nodes with the greatest increase in marginal value in a warm winter were those with the highest forage availability (Figure 3b). Only 4.8% of all nodes (Figure 3a, lower right quadrant) had a positive impact in a cold winter but a negative impact in a warm winter.

### 3.4 Net effects of node removal

Each node represents 0.014% of the total study area (6950 nodes). To understand the marginal value of a node in proportion to its size, a node in the 90th percentile of impact on survival (strong positive impact) in any year increased overall dabbling duck survival by 0.0015% (240 birds) on average and increased total DUD by 0.0005% (26,232 DUD). This amount represents only 10.6% (i.e., 0.0015/0.014) and 3.8% of the expected impact of that node, respectively, if its impact was proportional to its size, that the loss even of a strongly contributing single node was compensated in large part by the availability of other nodes. Similarly, a node with strong negative impacts (10th percentile) decreased survival of dabbling ducks to the spring on average by 10.5% as much as would be expected given node size (236 birds) and decreased total DUD by 5.5% as much as expected (38,048 DUD).

### 3.5 Marginal values of NWR nodes

**FIGURE 4:**
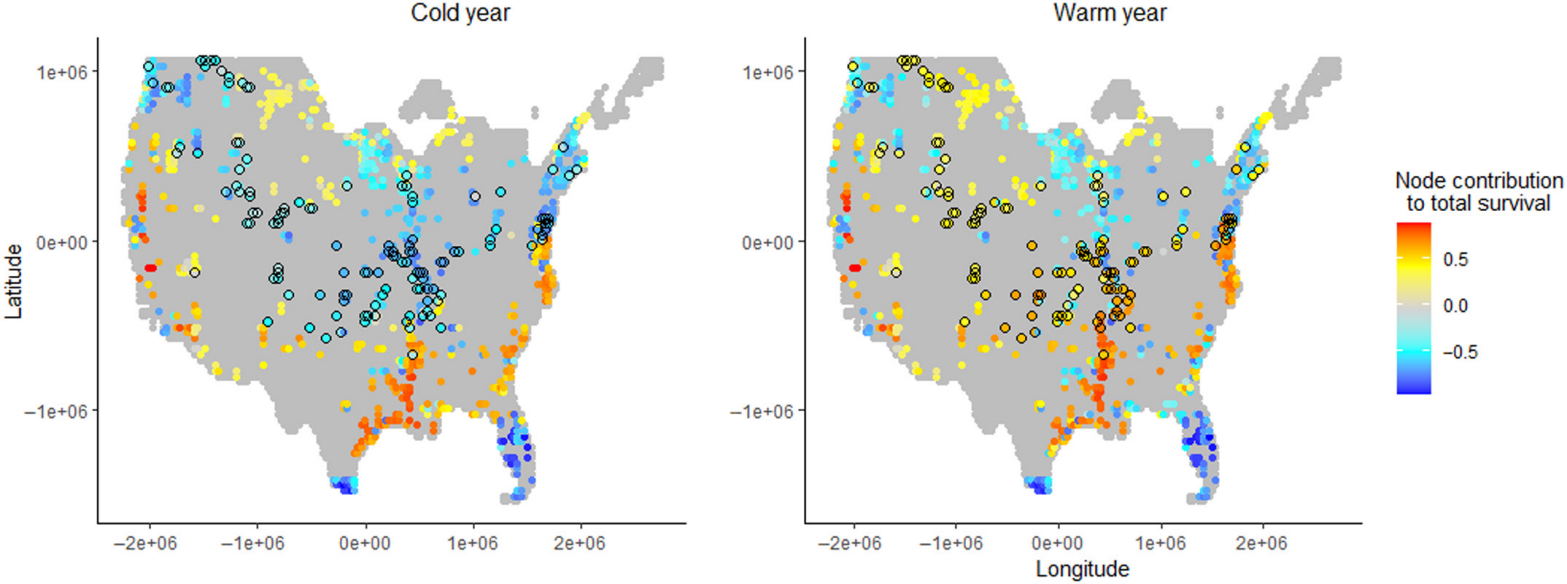
Node marginal value for total annual dabbling duck survival for nodes containing NWRs. Color shows contributions in a relatively cold (left) and warm (right) year. Nodes that differ in the sign of their contributions between years are shown with black outlines. To better display the variation among nodes while reducing the influence of extreme values, positive values were log‐transformed. For negative values, the absolute value was log‐transformed, and the negative sign was then restored. Positive and negative values were each then scaled proportionately to each other for easy comparison. For a map of nodes that switch from negative to positive values in a warm (vs. cold) year, refer to Appendix 8. All maps use Albers equal area conical projection centered on the contiguous United States.

The mean marginal values of the 20% of nodes containing NWRs (Figure 4) differed among years with different weather (Appendix 1; Figure 4). The proportion of nodes containing NWRs with a positive impact on SURV in a warm winter (56%) was higher than the proportion in a cold winter (45%), but each of these values was lower than the proportions for nodes that did not contain NWRs in the respective years (69% and 52%, respectively). These patterns also held for DUD (Appendix 7).

Spatially, NWR‐containing nodes shifting from negative to positive marginal value for survival in a warmer (vs. a colder) winter were primarily concentrated in the central United States, including along the central Mississippi Valley, as well as select locations in the Great Plains and Northwest (Figure 4; Appendix 8). In a cold winter, nodes containing NWRs did not differ from all other nodes in Δ SURV (*p* = .38, *t* = 0.89, df = 844) or Δ DUD (*p* = .22, *t* = 1.23, df = 847). However, in a warm winter, Δ SURV was lower in nodes containing NWRs (*p* < .01, *t* = 2.83, df = 840), as was Δ DUD (*p* < .01, *t* = 3.42, df = 843).

## 4 DISCUSSION

Annual weather variation has profound implications for the value of locations in supporting waterfowl migration. Using a continental‐scale energetics‐based model of daily dabbling duck movement (Aagaard et al., 2022) and node knockout simulations in the contiguous United States, we found that the marginal value of locations varied through space and time, with the Southeast, Mississippi Alluvial Valley, and California consistently among the most important (Figure 1). However, most locations on the southern, western, and the north‐central edges of the contiguous United States were detrimental to supporting waterfowl migration in a warmer winter, with a prominent east–west band running through the interior that increased in importance compared to a colder winter. Importantly, removal of habitat in some locations in some regions, especially the Great Lakes and central Great Plains, consistently decreased mortality in our simulations, indicating that there are possibly numerous sink habitats (Erwin, 2002) across the nation.

Impacts of node removal most influenced waterfowl populations at different times of year depending on the node removed. Southerly nodes predominantly reduced mortality in mid to late winter when weather severity was typically highest farther north. Some northerly nodes reduced mortality primarily during fall migration (Figure 2), whereas other northerly nodes increased mortality in spring migration, perhaps because they drew birds north early and thus exposed them to extreme weather (Lehikoinen et al., 2019; Newton, 2007). Exploring the conditions giving rise to these potential temporary or seasonal ‘ecological trap’ nodes would be valuable. Such traps have been explored in migrating passerine birds (Domer et al., 2021), but less so in waterfowl (Buderman et al., 2020). Such traps would be a concern, given apparent decreases in mallard populations, at least in the eastern United States, in recent years (Fink et al., 2022; Roberts et al., 2023).

Node importance is also not static among years because of varying weather. More northerly nodes, especially those in agricultural regions like the Great Plains and Midwest, were more likely to have a positive impact on duck survival in a warmer winter (Figure 1), which is important given predicted warming in the region throughout this century (Deser et al., 2012; Rawlins et al., 2016). Northward migration has begun earlier for many migratory bird species in recent decades (Lehikoinen et al., 2019), although the timing of spring northward movements can seldom be strongly predicted based on thaw and green‐up phenology in a given year (Wang et al., 2019). Furthermore, migration timing and forage types vary among dabbling duck species and our results are most relevant for mallards, the species for which the model was parameterized (Aagaard et al., 2022).

Intuitively, annual forage availability in a location affects its importance to supporting waterfowl migration (Figure 3; Lovvorn, 1989; Reinecke et al., 1989). But, in our simulations, nodes with relatively high forage can have strong negative marginal values for survival in a particular year, depending on geography. This counter‐intuitive result is probably because northerly nodes with large amounts of forage can cause birds to remain longer (or arrive earlier) in areas where there is increased risk of severe weather, increasing the risk of weather‐induced mortality (Trautman et al., 1939). These risks could differ for dabbling duck species that migrate earlier or later than mallards, but most have not been studied in sufficient detail to parameterize an equivalent model to quantify this risk. There is evidence from large‐scale citizen science data (eBird) that waterfowl shift winter and spring distributions in response to extreme weather (Masto et al., 2022), although the northerly ‘pull’ of the breeding ground appeared stronger than the southerly ‘push’ of these climatic events. Boos et al. (2007) found that winter mallard body condition in Europe did not relate to food availability or weather severity, indicating that the relationship between climate, forage, and survival is complex.

The many nodes containing lands protected as part of the NWR system are widely distributed across the contiguous United States (Figure 4) and as such broadly reflect the diversity of positive and negative node marginal values in our simulations. A higher proportion of these NWR‐containing nodes contributed positively in a warm winter compared to a cold winter, but the proportion of these nodes with positive contributions was lower than the proportion of all nodes contributing positively. This difference may reflect the relatively central and northerly distribution of many refuges, which on average occur outside of the belt of nodes in the Southeast and California that have the strongest positive contributions (with the notable exception of refuges along the lower Mississippi Valley and Gulf Coast). Substantial changes in the wintering bird communities on refuges are expected over the next three decades as climate changes (Wu et al., 2022). However, adaptive climate planning and management across the refuges of the NWR system is now common (Fischman et al., 2014) although specific climate planning for waterfowl remains rare. The information here (especially Appendix 1) can, for instance, aid refuge managers and biologists in identifying whether their refuge is likely to serve as a refugia under changing climate (i.e., those sets of reserves positively contributing in both cold and warm winters), as a welcomer to larger numbers of wintering waterfowl (i.e., northerly refuges positively contributing in warmer winters but not colder ones), or as a refuge likely to become less useful in supporting wintering waterfowl populations (i.e., southerly refuges weakly or negatively contributing in warmer winters). This insight could help the NWR system in identifying, respectively, areas where to conserve current habitat, promote adaptive habitat management actions, or acknowledge the direction of those changes and alter resource allocation accordingly.

Our simulations predict that a warmer future will likely result in increased marginal values of refuges in more central and northerly locations to duck survival, especially in the southern Midwest, Great Plains, and Northwest, and decreased marginal values in more southerly locations. In our simulations, loss of a single high‐quality node was largely compensated for by the remaining nodes, with much lower additional mortality as a result of node removal than would be expected given a node's area and baseline duck use. However, future land use change is unlikely to affect single nodes in isolation but may rather be pervasive across the landscape (Ordonez et al., 2014). In the case of agricultural shifts (Ramankutty et al., 2018), however, the outcomes may be mixed for dabbling ducks. Future work could use this duck migration model to simulate large‐scale scenarios based on projections of urban and suburban development, agricultural expansions, and crop shifts under climate scenarios to examine the range‐wide impacts on waterfowl populations.

A node's marginal value and the contributions of a refuge that it contains are not synonymous—our relatively coarse simulation (32 × 32 km grid) allowed us to uncover broad geographic patterns, but it does not allow us to assess the value of, for instance, a patch of high‐quality habitat (i.e., an NWR) within an otherwise unsuitable node. Refuges occur in increasingly fragmented and developed landscape matrices (Hamilton et al., 2013, 2015, 2016), although it is unclear how this fragmentation compares to other nodes away from the refuge system. Nevertheless, individual refuges may therefore have far higher (or lower) marginal values than our analyses indicate (Wauchope et al., 2022).

Our choices of single representative cold and warm winters helped us overcome challenges of computation time but mean that idiosyncrasies of those years may affect our results. Our warm winter, for example, was marginally colder than average in parts of the southern Rockies despite being warmer in other regions. This limitation may constrain our conclusions in that region, although this area is of relatively low importance for wintering ducks. Additionally, climate has direct impacts on forage availability and extent of surface water on the landscape, but for simplicity, our model assumes a static value of habitat across simulation years (Matchett & Fleskes, 2017; Reiter et al., 2018). A stochastic model incorporating full variability in, for example, habitats and uncertainty in model parameter values would better reflect the range of possible outcomes. Our relatively coarse spatial scale, necessitated by the same constraints, means that fine‐scale predictions of habitat use are best made with regional or local models (Beatty et al., 2017). Waterfowl movement data (Henry et al., 2016; McDuie et al., 2019) collected at appropriate scales would be useful for testing the assumptions of our model.

## 5 CONCLUSIONS

The proportion of nodes making positive contributions to duck survival increased considerably in a warmer winter, relative to a colder one. This switch is indicative of increasing value of many central and northerly habitat in the contiguous United States under warmer future climates (Deser et al., 2012). Many NWRs fall into this category and their value for dabbling ducks may increase. Our results highlight the geographic and temporal variability in habitat value, and the shifts that may occur in these values due to a changing climate.

## AUTHOR CONTRIBUTIONS

**Ryan C. Burner:** Conceptualization (equal); formal analysis (lead); methodology (equal); writing – original draft (lead); writing – review and editing (equal). **Benjamin D. Golas:** Formal analysis (supporting); methodology (equal); writing – review and editing (equal). **Kevin J. Aagaard:** Methodology (equal); writing – review and editing (equal). **Eric V. Lonsdorf:** Conceptualization (equal); writing – review and editing (equal). **Wayne E. Thogmartin:** Conceptualization (lead); funding acquisition (lead); methodology (equal); project administration (lead); writing – review and editing (equal).

## CONFLICT OF INTEREST STATEMENT

The authors have no competing interests to declare.

## Data Availability

Model code and details on climate and landscape information used in this study are available as supporting information in Aagaard et al. (2022).

## APPENDIX 1

Marginal values of nodes containing U.S. Fish and Wildlife Service (USFWS) units to dabbling duck total survival and DUD during the migration and winter seasons. Values are based on single‐node knockouts using the migration model of Aagaard et al. (2022). Table includes all 420 named USFWS units that occur primarily in the migration habitat zone of the contiguous United States, as defined in this study. Some units farther north are excluded. This marginal value analysis was run for a relatively cold winter (1957) and a relatively warm winter (2015). Values show change in survival (Δ SURV) and DUD (Δ DUD) when a node is present, relative to when it is removed. When a unit spanned multiple nodes, values are a weighted average of all node values, weighted by area of the unit in that node. The ‘survival quadrant’ value shows the relationship between node marginal value for survival in a cold and a warm winter; nodes can make a positive contribution in both years (Quadrant Q‐I; 40% of refuges), a negative contribution in both years (Q‐III; 40%), or a positive contribution only in a cold (Q‐IV; 5%) or a warm (Q‐II; 15%) year. Refer to Figure 3 for a visual representation of these quadrants. Unit total area was based on the ‘FWS National Realty Tracts’ shapefile of all tracts of NWR land from the U.S. Fish and Wildlife Service (USFWS; https://gis‐fws.opendata.arcgis.com/).

**Table:**

U.S. Fish and Wildlife Service unit name	Total area (km^2^)	Δ SURV (cold year)	Δ SURV (warm year)	Δ DUD (cold year)	Δ DUD (warm year)	Nodes (*n*)	Survival quadrant
Alamosa NWR	48.9	−35.5	40.0	−4923.8	108.8	1	II
Alligator River NWR	621.5	960.7	663.2	116,335.2	68,627.5	2	I
Amagansett NWR	0.2	−178.5	−110.9	−32,317.8	−22,351.0	1	III
Anaho Island NWR	2.2	102.3	68.5	13,315.2	3688.2	1	I
Anahuac NWR	157.5	1188.7	581.3	141,921.5	69,822.2	2	I
Ankeny NWR	11.3	−724.7	−703.3	−90,924.4	−106,991.0	1	III
Antioch Dunes NWR	0.2	−468.8	−653.0	−63,773.1	−108,041.8	1	III
Aransas NWR	372.5	791.8	117.7	88,700.8	12,231.4	5	I
Arapaho NWR	91.3	−12.0	13.7	−2768.6	−176.4	3	II
Archie Carr NWR	1.0	−1571.1	−2231.1	−205,524.7	−273,977.1	2	III
Arthur R. Marshall Loxahatchee NWR	587.9	−2477.2	−2447.0	−307,598.8	−305,718.8	1	III
Ash Meadows NWR	38.5	107.1	68.6	14,326.1	5342.5	4	I
Assabet River NWR	9.5	−288.7	−317.6	−50,017.4	−53,688.5	2	III
Atchafalaya NWR	63.8	1722.7	762.5	203,973.5	89,343.7	1	I
Attwater Prairie Chicken NWR	42.8	1490.4	629.1	177,616.4	77,669.9	3	I
Baca NWR	47.5	−10.9	23.5	−1682.2	163.6	2	II
Back Bay NWR	35.1	−769.7	−613.7	−109,608.0	−98,848.1	1	III
Balcones Canyonlands NWR	110.0	−108.0	−225.9	−14,098.5	−28,520.4	4	III
Bald Knob NWR	62.5	−135.2	94.0	−17,245.1	3518.3	1	II
Bamforth NWR	4.7	−2.2	9.6	−1110.0	145.9	1	II
Bandon Marsh NWR	3.7	146.8	81.7	19,644.3	−1467.9	1	I
Banks Lake NWR	12.1	175.1	26.7	19,799.8	−1695.8	1	I
Baskett Slough NWR	10.8	−165.3	−159.8	−16,706.2	−26,199.2	1	III
Bayou Cocodrie NWR	61.3	1460.5	733.0	186,367.0	94,397.6	1	I
Bayou Sauvage Urban NWR	103.3	22.7	−685.3	−4344.3	−94,948.5	1	IV
Bayou Teche NWR	44.4	1724.2	531.5	202,875.5	58,134.5	1	I
Bear Butte NWR	1.6	19.5	20.9	3492.4	2559.7	1	I
Bear Lake NWR	74.0	−28.9	0.0	−4553.7	−2078.5	2	II
Bear River Migratory BR	309.5	−363.3	−142.3	−49,947.6	−37,531.4	3	III
Bear Valley NWR	17.1	−243.6	−355.5	−28,341.0	−71,270.8	1	III
Benton Lake NWR	50.2	−8.4	46.8	−626.9	14,023.4	2	II
Big Boggy NWR	18.2	519.2	193.6	62,500.2	23,968.9	1	I
Big Branch Marsh NWR	78.4	0.1	−552.7	−6694.3	−77,564.8	2	IV
Big Lake NWR	44.2	−291.7	−6.4	−32,875.7	−5827.2	2	III
Big Muddy National FWR	74.3	−305.1	−59.1	−45,381.7	−23,150.5	10	III
Big Oaks NWR	204.3	−160.0	95.6	−23,991.5	−929.6	1	II
Big Stone NWR	47.0	−26.5	−44.4	−6608.7	−5528.2	2	III
Bill Williams River NWR	24.3	153.7	84.3	18,661.8	9741.5	2	I
Billy Frank Jr. Nisqually NWR	24.9	−242.6	−157.5	−36,788.7	−22,312.5	2	III
Bitter Creek NWR	14.4	97.2	54.7	11,704.7	5663.8	1	I
Bitter Lake NWR	100.6	187.5	85.0	24,339.8	8395.0	2	I
Black Bayou Lake NWR	21.3	−827.9	−1103.7	−102,823.4	−140,730.8	1	III
Black Coulee NWR	5.3	22.3	44.7	3335.0	12,606.1	2	I
Blackbeard Island NWR	22.6	384.2	94.9	42,779.6	3187.5	1	I
Blackwater NWR	136.2	−131.9	301.7	−15,495.8	30,838.7	6	II
Block Island NWR	0.5	−15.7	31.7	−3638.5	2663.2	1	II
Blue Ridge NWR	3.7	290.2	194.7	36,939.1	18,748.8	1	I
Bogue Chitto NWR	147.2	477.7	176.1	59,565.8	19,617.6	2	I
Bombay Hook NWR	62.3	−199.2	253.9	−25,303.1	20,452.4	3	II
Bon Secour NWR	29.3	61.3	−99.3	4359.4	−16,384.6	2	IV
Bond Swamp NWR	26.7	83.6	−82.4	7938.0	−18,721.3	2	IV
Bosque Del Apache NWR	231.7	490.8	311.7	63,426.9	36,860.7	2	I
Bowdoin NWR	62.9	15.5	37.9	3497.8	10,011.1	1	I
Boyer Chute NWR	16.1	−208.2	−138.6	−36,631.9	−24,325.7	1	III
Brazoria NWR	190.3	96.6	−433.9	10,267.7	−54,240.0	4	IV
Breton NWR	125.1	235.1	50.9	26,284.0	4260.5	2	I
Browns Park NWR	32.3	−1.9	19.2	−873.2	561.1	2	II
Buenos Aires NWR	111.3	20.4	9.6	2448.5	1113.4	3	I
Cabeza Prieta NWR	5303.6	7.1	3.2	836.1	374.6	4	I
Cache River NWR	298.0	46.2	557.4	8738.2	61,066.8	5	I
Caddo Lake NWR	31.4	−85.9	−98.8	−12,041.2	−16,708.9	2	III
Cahaba River NWR	14.7	75.5	−24.9	8002.0	−9831.5	1	IV
Caloosahatchee NWR	0.1	−1863.4	−2453.6	−239,867.9	−305,204.2	1	III
Camas NWR	43.7	−89.3	−38.5	−13,889.3	−5266.3	2	III
Cameron Prairie NWR	65.7	1179.3	556.8	140,420.3	66,538.5	2	I
Canaan Valley NWR	68.8	1.3	78.5	348.9	8642.4	1	I
Cape May NWR	47.4	−193.9	74.2	−29,114.2	−6010.9	3	II
Cape Meares NWR	0.6	11.2	10.8	3814.4	−1585.4	1	I
Cape Romain NWR	139.7	512.4	200.9	58,905.7	9096.6	3	I
Carolina Sandhills NWR	185.8	252.6	304.2	26,468.5	30,079.0	2	I
Castle Rock NWR	0.1	262.4	176.7	33,466.4	9005.0	1	I
Cat Island NWR	37.8	1336.3	728.0	168,415.1	91,477.7	1	I
Catahoula NWR	102.3	1012.7	599.9	133,484.7	79,257.6	2	I
Cedar Island NWR	58.0	334.2	212.1	39,191.9	19,686.7	4	I
Cedar Keys NWR	3.4	225.6	−82.1	21,658.6	−14,947.8	1	IV
Cedar Point NWR	10.5	−322.8	−280.4	−57,786.7	−51,146.7	1	III
Charles M. Russell NWR	3003.4	17.2	30.6	3053.3	7150.6	16	I
Chassahowitzka NWR	105.2	−561.8	−1088.9	−80,509.3	−148,545.3	1	III
Chautauqua NWR	26.9	−238.6	−173.4	−47,202.0	−34,277.1	3	III
Cherry Valley NWR	22.0	−349.8	−215.5	−57,413.1	−36,494.2	1	III
Chickasaw NWR	108.0	−4.3	570.9	2721.3	63,894.3	3	II
Chincoteague NWR	13.3	387.9	401.6	46,077.5	43,960.8	4	I
Choctaw NWR	16.1	434.3	279.3	52,825.7	31,914.5	1	I
Cibola NWR	61.5	886.4	515.1	107,539.8	61,835.7	3	I
Clarence Cannon NWR	15.1	−239.5	142.7	−37,620.7	114.9	2	II
Clarks River NWR	37.8	−254.8	138.7	−31,235.3	2569.8	1	II
Clear Lake NWR	45.3	15.9	−57.9	7133.5	−20,135.2	1	IV
Coachella Valley NWR	14.6	23.1	−42.8	82.5	−8716.3	2	IV
Cokeville Meadows NWR	32.2	−7.5	16.8	−1339.0	271.7	2	II
Cold Springs NWR	8.2	−597.9	−275.4	−84,511.7	−24,551.2	1	III
Coldwater River NWR	10.2	751.7	801.6	98,605.5	98,470.1	1	I
Columbia NWR	120.2	−458.9	−167.3	−63,949.6	−10,712.0	4	III
Colusa NWR	16.6	1632.6	1160.9	207,253.6	89,570.6	1	I
Conboy Lake NWR	29.3	−20.6	10.2	−3010.9	2725.0	2	II
Rep. Lester Wolff Oyster Bay NWR	13.4	−250.9	−270.9	−42,175.1	−46,453.7	1	III
Conscience Point NWR	0.2	−178.5	−110.9	−323,17.8	−22,351.0	1	III
Crab Orchard NWR	183.0	−353.7	−254.8	−50,518.2	−47,975.5	1	III
Crane Meadows NWR	8.8	−4.2	−42.8	−874.2	−4756.6	1	III
Creedman Coulee NWR	11.0	24.3	48.2	3309.4	14,299.8	1	I
Crescent Lake NWR	185.8	−12.4	12.9	−3556.3	−358.4	3	II
Crocodile Lake NWR	27.3	−776.3	−869.9	−97,113.2	−108,092.3	1	III
Cross Creeks NWR	35.7	−25.0	245.8	−380.4	24,499.1	2	II
Crystal River NWR	0.6	−814.6	−1304.8	−110,939.5	−175,122.0	2	III
Currituck NWR	32.4	214.8	211.7	25,052.3	22,462.4	2	I
Cypress Creek NWR	60.6	−301.9	34.0	−39,136.7	−11,082.8	2	II
D ‘Arbonne’ NWR	71.4	−827.9	−1103.7	−102,823.4	−140,730.8	1	III
Dahomey NWR	39.6	349.5	359.4	51,925.5	50,479.4	1	I
Dale Bumpers White River NWR	617.6	408.5	772.6	56,368.6	93,915.3	5	I
Deep Fork NWR	42.7	−51.5	29.4	−8147.3	−2563.2	4	II
Deer Flat NWR	47.1	−709.4	−424.5	−92,100.5	−70,927.4	7	III
Delevan NWR	23.6	1963.2	1360.5	248,880.0	111,938.1	2	I
Delta NWR	81.9	1001.9	351.2	115,716.7	39,312.5	2	I
Desert National WRg	21,594.4	8.4	5.1	1109.9	290.4	8	I
Desoto NWR	33.8	−208.2	−138.6	−36,631.9	−24,325.7	1	III
Detroit River International WR	22.0	−415.5	−465.1	−70,947.6	−79,740.4	2	III
D. Edwards San Francisco Bay NWR	118.3	−477.7	−534.6	−64,959.3	−77,548.9	1	III
Driftless Area NWR	4.9	−41.9	−26.3	−10,355.4	−6799.3	6	III
Eastern Neck NWR	8.5	−244.7	337.6	−32,563.6	25,106.4	2	II
Eastern Shore Of Virginia NWR	5.4	467.9	468.3	55,911.5	51,722.6	1	I
Edwin B. Forsythe NWR	166.9	−418.3	−321.3	−64,164.9	−63,397.4	3	III
Egmont Key NWR	1.3	−18.4	−85.8	−4134.8	−11,550.3	1	III
Elizabeth Alexandra Morton NWR	0.7	−178.5	−110.9	−32,317.8	−22,351.0	1	III
Elizabeth Hartwell Mason Neck NWR	9.2	−550.0	−595.1	−83,338.9	−97,001.8	1	III
Ellicott Slough NWR	0.8	53.6	−116.6	1841.3	−39,202.5	1	IV
Emiquon NWR	10.6	−219.5	−134.9	−45,238.7	−28,949.1	2	III
Erie NWR	36.5	−100.8	21.6	−17,562.7	2190.7	1	II
Ernest F. Hollings Ace Basin NWR	49.0	787.9	465.9	95,739.9	44,774.4	2	I
Eufaula NWR	14.9	745.8	454.2	87,332.7	47,073.3	1	I
Everglades Headwaters NWR & CA	41.8	−497.3	−1117.6	−74,690.4	−140,093.2	6	III
Fallon NWR	73.1	264.6	172.8	33,478.8	3860.0	1	I
Farallon Islands NWR	0.5	126.3	73.9	15,102.0	6967.1	1	I
Featherstone NWR	1.4	−550.0	−595.1	−83,338.9	−97,001.8	1	III
Felsenthal NWR	283.7	−62.1	−68.7	−6731.1	−9647.3	3	III
Fern Cave NWR	0.8	−611.6	−657.3	−81,461.9	−96,059.7	1	III
Fish Springs NWR	16.3	37.6	27.4	5311.6	741.0	1	I
Fisherman Island NWR	7.8	456.7	456.7	54,408.4	50,190.8	2	I
Flint Hills NWR	75.1	−216.6	55.5	−33,276.5	−7196.5	3	II
Florida Panther NWR	107.2	−521.5	−564.2	−66,942.5	−71,939.2	2	III
Fort Niobrara NWR	77.3	20.6	35.1	1932.1	3687.1	1	I
Fox River NWR	4.0	−37.6	−67.9	−8549.7	−7191.2	2	III
Franklin Island NWR	0.1	30.3	49.7	5748.1	13,023.5	1	I
Franz Lake NWR	2.4	−34.7	−12.1	−2363.2	−3425.6	1	III
Grand Bay NWR	42.1	275.9	−83.1	29,189.4	−16,044.9	2	IV
Grand Cote NWR	2.3	1027.5	457.2	133,518.9	60,037.4	1	I
Grass Lake NWR	16.4	11.5	15.0	1367.5	4657.2	1	I
Gravel Island NWR	0.1	41.0	25.0	4274.3	6555.7	1	I
Grays Harbor NWR	5.7	−103.3	−30.3	−12,770.3	−1002.1	1	III
Grays Lake NWR	68.9	−11.1	16.3	−2077.2	111.6	1	II
Great Bay NWR	4.7	−171.0	−196.6	−32,892.9	−32,927.8	3	III
Great Dismal Swamp NWR	461.7	−626.1	−608.8	−90,527.3	−93,215.1	2	III
Great Meadows NWR	14.9	−277.7	−306.0	−48,519.5	−52,806.1	2	III
Great River NWR	47.9	−444.4	−192.9	−66,390.1	−45,615.1	5	III
Great Swamp NWR	31.8	−350.9	−380.2	−58,164.1	−64,438.3	1	III
Great Thicket NWR	1.1	−123.0	−51.4	−22,512.6	−11,474.6	3	III
Great White Heron NWR	547.6	−977.7	−933.4	−120,951.4	−116,532.8	4	III
Green Bay NWR	7.3	29.5	20.8	3399.9	4701.5	3	I
Green River NWR & CPA	2.7	−854.9	−648.0	−113,655.5	−105,793.1	1	III
Gregory County WPA	1.3	6.2	41.6	−1097.7	4836.0	1	I
Grulla NWR	13.1	604.7	331.8	81,249.6	40,458.9	1	I
Guadalupe‐Nipomo Dunes NWR	10.1	991.7	492.0	117,122.1	45,034.1	1	I
Hackmatack NWR	0.8	−309.3	−396.0	−55,812.9	−65,642.4	3	III
Hagerman NWR	45.7	85.2	148.5	12,172.7	18,192.2	1	I
Hailstone NWR	3.6	7.1	8.7	1269.4	3199.0	1	I
Handy Brake NWR	2.0	227.5	284.6	34,120.0	37,524.4	1	I
Hanford Reach NM/Saddle Mtn NWR	661.7	−294.3	−103.1	−41,050.7	−5367.4	3	III
Harbor Island NWR	3.0	26.5	11.9	6085.9	3201.2	1	I
Harris Neck NWR	11.4	480.3	136.8	54,301.8	6590.2	1	I
Hart Mountain National AR	340.2	39.2	45.3	7092.2	−2007.0	4	I
Hatchie NWR	46.2	−216.2	−8.7	−25,178.1	−6448.5	2	III
Havasu NWR	157.7	389.0	253.3	48,620.1	25,743.1	2	I
Hewitt Lake NWR	2.9	15.5	37.9	3497.8	10,011.1	1	I
Hillside NWR	62.7	696.8	715.9	94,118.4	92,253.4	1	I
Hobe Sound NWR	4.4	−3217.8	−4153.0	−409,555.5	−506,163.9	1	III
Holla Bend NWR	24.6	15.1	182.8	2209.9	17,633.9	1	I
Holt Collier NWR	6.2	1009.0	1082.2	132,316.7	134,170.2	1	I
Hopper Mountain NWR	9.5	416.7	227.9	50,109.0	21,692.4	1	I
Horicon NWR	89.0	−134.7	−160.2	−29,241.1	−29,212.6	2	III
Humboldt Bay NWR	14.7	160.7	93.7	19,348.4	−608.8	2	I
Hutton Lake NWR	8.1	−9.0	2.6	−2134.0	−810.6	1	II
Imperial NWR	119.1	304.5	171.4	36,795.1	20,361.4	2	I
Iroquois NWR	43.8	−102.8	−62.8	−16,999.0	−2820.8	1	III
Island Bay NWR	0.1	−1307.6	−1899.0	−171,707.8	−23,6458.6	1	III
J. N. Ding Darling NWR	26.6	−2633.4	−2581.2	−321,099.8	−315,674.6	1	III
James River NWR	18.7	87.2	262.7	7078.4	26,178.9	1	I
John H. Chafee NWR	2.3	−272.8	−236.0	−47,418.9	−43,201.2	1	III
John Heinz NWR At Tinicum	4.1	−495.6	−532.6	−75,427.2	−86,191.0	1	III
John W. & Louise Seier NWR	9.7	22.6	36.9	2305.6	4287.7	1	I
Julia Butler Hansen RCD	23.9	−209.2	−81.8	−25,938.5	−6791.6	3	III
Kankakee NWR & CA	0.3	−170.5	−80.5	−31,389.3	−14,745.4	1	III
Karl E. Mundt NWR	5.7	6.2	41.6	−1097.7	4836.0	1	I
Kern NWR	45.5	3075.1	1743.9	370,969.2	190,743.4	2	I
Key Cave NWR	4.3	−82.6	76.2	−9000.5	5953.9	1	II
Key West NWR	852.5	−441.0	−442.3	−55,239.8	−55,643.5	1	III
Kirwin NWR	43.6	−223.7	140.9	−35,609.5	403.1	1	II
Klamath Marsh NWR	177.1	89.4	41.3	13,224.3	1165.8	2	I
Kofa NWR	2689.2	8.7	4.5	1012.7	459.7	4	I
Lacassine NWR	124.2	1319.0	530.8	156,460.3	63,407.4	4	I
Lacreek NWR	40.1	5.7	17.2	23.7	1529.4	2	I
Laguna Atascosa NWR	412.7	−1251.9	−1784.9	−154,590.9	−217,117.4	7	III
Lake Andes NWR	23.1	2.5	34.5	−1437.3	4059.0	3	I
Lake Ilo NWR	16.2	27.2	2.7	3638.3	3292.7	2	I
Lake Isom NWR	7.3	−243.5	212.9	−31,235.2	5919.5	1	II
Lake Mason NWR	68.0	14.5	18.8	2343.4	3831.9	2	I
Lake Ophelia NWR	70.6	1619.5	1077.9	209,847.2	139,339.8	2	I
Lake Patricia NWR	3.2	35.8	10.2	4835.6	4565.1	1	I
Lake Thibadeau NWR	15.9	24.3	48.2	3309.4	14,299.8	1	I
Lake Wales Ridge NWR	7.7	−2545.2	−3679.6	−335,065.3	−452,984.6	3	III
Lake Woodruff NWR	87.3	−235.4	−707.1	−40,083.3	−97,000.1	2	III
Lake Zahl NWR	15.7	−8.3	−21.5	3588.8	696.2	2	III
Lamesteer NWR	3.3	23.6	9.0	2930.5	3975.8	1	I
Las Vegas NWR	35.3	29.8	61.3	4091.7	4731.3	1	I
Lee Metcalf NWR	11.4	−41.5	−8.2	−6335.4	2401.7	2	III
Leslie Canyon NWR	66.6	12.7	5.1	1471.7	496.2	2	I
Lewis & Clark NWR	52.9	−208.4	−74.3	−25,149.1	−5490.6	1	III
Little River NWR	48.1	110.9	138.1	15,899.1	17,918.4	1	I
Little Sandy NWR	15.5	−127.2	−134.4	−15,996.3	−20,184.0	1	III
Loess Bluffs NWR	30.1	−196.7	−15.1	−41,062.4	−13,747.6	1	III
Logan Cave NWR	0.5	−120.5	−111.1	−19,074.9	−21,378.1	1	III
Lost Trail NWR	36.2	−1.0	23.5	214.5	5970.2	1	II
Lower Hatchie NWR	57.1	−79.2	350.8	−7228.5	36,185.2	2	II
Lower Klamath NWR	209.2	157.6	64.4	23,237.4	−5086.8	2	I
Lower Rio Grande Valley NWR	407.0	−1454.1	−1750.3	−177,795.1	−213,236.2	14	III
Lower Suwannee NWR	210.5	291.2	−111.6	27,864.7	−20,355.1	3	IV
Mackay Island NWR	35.3	−766.8	−611.3	−109,204.3	−98,486.2	2	III
Malheur NWR	761.2	−12.6	6.7	582.9	−4693.2	5	II
Mandalay NWR	18.7	1624.6	619.5	189,530.6	69,927.5	1	I
Marais Des Cygnes NWR	30.8	−247.3	63.0	−38,420.3	−8319.5	1	II
Marin Islands NWR	1.9	179.1	28.7	18,388.9	−14,628.8	1	I
Martin NWR	17.3	196.8	296.5	22,196.1	33,937.2	2	I
Mashpee NWR	1.4	−151.4	−131.1	−26,719.1	−24,130.2	2	III
Massasoit NWR	0.8	−351.3	−278.8	−58,261.8	−48,428.7	1	III
Mathews Brake NWR	9.6	170.3	143.3	30,031.7	20,058.3	1	I
Matlacha Pass NWR	2.2	−2366.3	−2611.4	−293,306.1	−320,745.1	2	III
Mattamuskeet NWR	201.9	572.6	333.3	69,652.9	31,131.5	3	I
Maxwell NWR	14.7	−25.2	87.0	−3636.3	3972.2	1	II
Mcfaddin NWR	271.7	−435.3	−1020.1	−55,108.1	−127,152.1	3	III
Mckay Creek NWR	7.4	−254.1	−95.4	−34,178.4	−15,793.3	1	III
Mclean NWR	3.1	20.3	18.4	6093.1	5981.1	1	I
Mcnary NWR	64.1	−486.9	−325.4	−72,687.0	−33,315.1	6	III
Medicine Lake NWR	127.5	4.6	9.4	2779.0	5234.6	3	I
Merced NWR	15.5	1241.4	731.6	153,639.2	51,471.3	1	I
Meredosia NWR	15.0	−333.5	84.0	−48,327.2	−7782.2	1	II
Merritt Island NWR	144.8	61.2	−299.6	−17.3	−42,662.9	2	IV
Michigan Islands NWR	3.4	19.5	7.2	2462.7	2736.0	7	I
Middle Mississippi River NWR	33.2	−244.9	152.3	−32,144.9	1949.4	5	II
Mille Lacs NWR	0.00	24.9	16.5	4519.2	4406.8	1	I
Mingo NWR	87.8	−49.5	211.7	−5630.2	17,525.6	2	II
Minidoka NWR	99.1	−131.6	−26.8	−20,025.6	−9358.5	2	III
Minnesota Valley NWR	59.8	−169.4	−239.2	−30,819.5	−40,505.9	5	III
Mississippi Sandhill Crane NWR	53.6	−6.7	−343.2	−6287.3	−49,094.9	1	III
Moapa Valley NWR	0.5	27.8	15.2	3828.2	489.3	1	I
Modoc NWR	28.9	310.3	205.9	41,038.1	9040.3	1	I
Monomoy NWR	32.2	−6.3	60.6	−2532.3	6140.4	2	II
Monte Vista NWR	59.7	−10.3	34.4	−1416.8	581.0	1	II
Montezuma NWR	40.5	−109.8	−69.4	−16,580.0	−585.8	1	III
Moody NWR	14.2	608.7	295.1	73,225.1	35,723.1	1	I
Morgan Brake NWR	30.6	569.0	576.9	78,558.3	74,724.6	2	I
Mortenson Lake NWR	10.4	−4.4	26.4	−1810.7	1255.0	1	II
Mountain Longleaf NWR	36.5	−225.9	−366.0	−31,637.0	−55,156.2	1	III
Muleshoe NWR	26.5	127.9	18.6	20,785.9	2439.9	2	I
Muscatatuck NWR	31.7	−330.4	61.9	−46,093.0	−8996.6	2	II
Nansemond NWR	1.7	−828.3	−840.4	−119,299.9	−127,083.9	1	III
Nantucket NWR	0.1	40.3	113.9	5437.8	15,072.4	1	I
National ER	89.8	−17.8	−0.2	−2880.8	−1627.2	2	III
National Key DR	328.5	−436.0	−472.2	−55,126.2	−59,328.3	3	III
Neal Smith NWR	23.1	−153.5	−31.0	−34,225.3	−12,809.9	1	III
Necedah NWR	77.3	40.3	14.8	4239.0	7196.0	2	I
Neches River NWR	29.3	338.7	79.8	43,807.7	9302.1	2	I
Nestucca Bay NWR	4.8	−24.7	−30.4	−993.6	−8214.7	1	III
Ninepipe NWR	8.4	−80.0	−9.1	−13,226.1	5512.4	1	III
Ninigret NWR	3.6	−272.8	−236.0	−47,418.9	−43,201.2	1	III
Nomans Land Island NWR	2.5	−8.0	44.1	−1233.5	5946.0	1	II
North Platte NWR	11.8	−48.3	−35.0	−8714.0	−8818.4	2	III
Occoquan Bay NWR	2.6	−550.0	−595.1	−83,338.9	−97,001.8	1	III
Ohio River Islands NWR	12.9	−169.9	−71.2	−26,199.6	−19,994.9	9	III
Okefenokee NWR	1650.7	434.7	188.0	49,824.1	15,707.2	6	I
Optima NWR	17.6	−149.8	238.2	−19,891.8	11,242.3	1	II
Oregon Islands NWR	2.1	48.2	23.3	6572.7	−3034.6	8	I
Ottawa NWR	32.4	−460.7	−381.2	−79,280.4	−66,959.2	3	III
Ouray NWR	48.9	−14.8	40.6	−3169.9	1278.0	2	II
Overflow NWR	55.0	183.5	219.6	25,171.7	26,559.8	1	I
Oxbow NWR	6.9	−290.4	−319.4	−50,248.8	−53,832.2	1	III
Ozark Cavefish NWR	0.2	−66.0	20.1	−10,166.9	−4596.6	2	II
Ozark Plateau NWR	17.7	−12.2	12.8	−2161.9	−555.7	6	II
Pablo NWR	10.0	−43.2	32.3	−5880.1	12,310.4	1	II
Pahranagat NWR	18.8	38.7	24.9	5143.2	1601.0	2	I
Panther Swamp NWR	165.2	1119.0	1189.0	145,497.6	149,405.4	3	I
Parker River NWR	18.5	−105.5	−110.9	−20,662.6	−18,641.3	2	III
Passage Key NWR	0.3	−18.4	−85.8	−4134.8	−11,550.3	1	III
Pathfinder NWR	68.2	7.1	13.7	631.8	1007.7	2	I
Patoka River NWR	44.3	−330.9	101.1	−47,087.5	−6404.0	3	II
Patuxent RR	51.9	−589.3	−588.3	−87,913.4	−98,115.5	2	III
Pea Island NWR	20.2	286.1	149.4	33,051.6	11,434.5	2	I
Pee Dee NWR	35.0	182.3	181.0	18,369.2	16,209.2	1	I
Pelican Island NWR	21.9	−3448.5	−4719.8	−443,573.9	−574,802.9	1	III
Piedmont NWR	160.0	251.3	182.6	31,770.2	20,104.6	1	I
Pierce NWR	1.6	−34.7	−12.1	−2363.2	−3425.6	1	III
Pilot Knob NWR	0.4	−23.4	11.4	−4109.7	−1773.0	1	II
Pinckney Island NWR	16.4	−368.9	−1076.8	−60,077.5	−155,558.6	3	III
Pine Island NWR	2.6	−2218.7	−2452.1	−275,533.8	−301,470.1	3	III
Pinellas NWR	1.6	−2454.4	−3248.3	−310,087.1	−396,921.1	2	III
Pixley NWR	30.0	3060.1	1756.7	368,731.4	192,359.9	1	I
Plum Tree Island NWR	12.4	−726.2	−853.7	−105,718.8	−125,483.9	2	III
Pocosin Lakes NWR	465.1	897.7	618.0	108,242.3	63,427.5	4	I
Pond Creek NWR	24.9	71.7	85.6	9945.3	10,650.3	1	I
Pond Island NWR	0.1	16.4	61.9	1893.5	13,056.5	1	I
Port Louisa NWR	97.2	−156.8	−44.0	−33,557.5	−12,809.8	5	III
Presquile NWR	5.2	135.0	339.4	13,029.2	35,192.8	2	I
Prime Hook NWR	41.0	−331.9	77.6	−46,718.7	−10,666.0	3	II
Quivira NWR	89.7	−251.7	159.1	−37,544.6	7370.1	1	II
Rachel Carson NWR	23.1	−77.8	−86.0	−16,227.0	−14,314.6	3	III
Rappahannock River Valley NWR	39.4	213.3	493.4	25,678.4	58,950.0	3	I
Red River NWR	63.3	699.3	302.0	89,412.0	38,623.5	6	I
Red Rock Lakes NWR	346.4	−5.4	10.4	−683.6	929.4	4	II
Reelfoot NWR	42.2	−243.5	212.9	−31,235.2	5919.5	1	II
Ridgefield NWR	20.2	−597.7	−364.2	−78,632.1	−41,729.3	1	III
Rio Mora NWR & CA	17.1	23.3	33.0	3084.9	2339.6	1	I
Roanoke River NWR	86.2	628.3	371.2	71,466.3	29,930.1	2	I
Rocky Flats NWR	21.2	−123.1	−38.3	−18,233.6	−13,705.3	1	III
Rocky Mountain Arsenal NWR	64.7	−948.6	−818.8	−126,208.9	−122,393.1	2	III
Rocky Mountain Front CA	146.7	4.0	29.0	531.7	8488.7	4	I
Ruby Lake NWR	161.7	26.5	26.2	4632.8	140.7	2	I
Sabine NWR	505.2	1237.7	565.7	146,805.1	67,307.7	2	I
Sachuest Point NWR	1.0	−114.4	−78.7	−21,577.9	−17,359.5	1	III
Sacramento NWR	44.2	1824.8	1263.5	231,506.7	106,463.6	2	I
Sacramento River NWR	46.8	1229.2	832.7	156,969.7	60,404.1	3	I
Salinas River NWR	1.5	−164.5	−318.4	−26,069.0	−63,838.0	1	III
Salt Plains NWR	130.1	−238.6	276.3	−30,245.0	10,337.8	4	II
Sam D. Hamilton Noxubee NWR	195.6	369.1	293.4	46,456.2	32,376.3	1	I
San Andres NWR	0.01	52.2	17.8	6286.3	947.1	1	I
San Bernard NWR	218.5	803.8	206.2	95,430.0	24,922.4	8	I
San Bernardino NWR	9.6	25.5	11.8	3023.6	1254.0	1	I
San Diego Bay NWR	10.5	−424.1	−487.7	−55,106.1	−61,761.0	2	III
San Diego NWR	50.4	−247.0	−302.1	−34,407.7	−40,554.6	2	III
San Joaquin River NWR	47.0	−22.0	−354.4	−5338.2	−84,776.3	2	III
San Luis NWR	72.1	1241.4	731.6	153,639.2	51,471.3	1	I
San Luis Valley CA	0.6	−2.6	17.2	−528.8	194.8	1	II
San Pablo Bay NWR	82.9	−492.3	−572.1	−64,283.0	−98,543.1	1	III
Sand Lake NWR	79.9	−3.2	−43.7	1024.3	−1503.3	2	III
Santa Ana NWR	8.5	−1821.3	−2191.3	−222,661.3	−267,705.2	1	III
Santee NWR	52.1	463.4	98.3	52,600.8	−4565.9	1	I
Sauta Cave NWR	1.1	8.4	205.5	1178.3	21,031.2	1	I
Savannah NWR	128.0	−319.4	−1014.6	−53,907.9	−147,550.6	4	III
Seal Beach NWR	4.0	−370.3	−419.2	−49,645.0	−54,679.9	1	III
Seal Island NWR	0.5	18.8	34.2	2231.8	5960.3	1	I
Seatuck NWR	0.9	−258.0	−282.2	−43,787.0	−48,981.3	1	III
Seedskadee NWR	105.2	−10.5	15.3	−1944.8	−85.0	2	II
Sequoyah NWR	84.7	0.3	160.1	−326.0	13,562.2	1	I
Sevilleta NWR	922.1	398.9	255.7	51,487.7	29,869.5	3	I
Shawangunk Grasslands NWR	2.4	−376.9	−339.4	−63,623.2	−57,549.0	1	III
Sheldon NWR	132.7	28.1	18.4	4008.4	303.0	4	I
Shell Keys NWR	0.02	421.8	111.2	48,789.3	10,917.9	1	I
Sherburne NWR	124.1	−70.4	−107.6	−12,413.0	−15,042.2	2	III
Shiawassee NWR	41.3	−413.5	−511.1	−71,342.1	−84,407.4	2	III
Siletz Bay NWR	2.3	−33.4	−36.4	−2801.3	−8075.4	1	III
Sonny Bono Salton Sea NWR	149.5	1749.4	1003.2	211,620.5	120,996.7	2	I
St. Catherine Creek NWR	100.3	1652.4	1049.9	208,530.7	132,436.8	2	I
St. Johns NWR	26.0	−437.5	−1016.0	−69,351.9	−130,866.3	2	III
St. Marks NWR	337.6	431.0	−19.5	46,074.1	−10,054.4	3	IV
St. Vincent NWR	49.3	378.2	38.9	41,601.9	28.7	2	I
Steigerwald Lake NWR	5.3	−635.3	−584.6	−86,554.9	−87,435.3	1	III
Stewart B. Mckinney NWR	4.1	−149.8	−131.4	−27,594.3	−25,425.3	6	III
Stewart Lake NWR	2.6	1.9	−38.9	394.2	−985.1	1	IV
Stillwater NWR	20.8	256.2	167.1	32,548.3	4234.2	2	I
Stone Lakes NWR	25.9	270.8	−46.5	29,456.1	−82,747.8	1	IV
Sunburst Lake NWR	1.3	13.8	−17.0	2497.2	346.9	1	IV
Supawna Meadows NWR	14.2	−952.6	−1031.0	−13,9054.0	−161,774.9	1	III
Susquehanna NWR	0.01	−428.1	−52.2	−61,671.3	−32,662.0	1	III
Sutter NWR	10.6	1956.8	1392.4	247,858.0	111,050.4	2	I
Swan Lake NWR	44.7	−305.3	102.6	−47,367.9	−8345.8	2	II
Swan River NWR	7.8	−32.6	35.2	−4404.5	12,011.9	1	II
Swanquarter NWR	67.3	605.5	366.5	73,474.6	35,123.1	2	I
Tallahatchie NWR	16.9	460.0	460.7	64,050.4	57,570.9	3	I
Target Rock NWR	0.3	−250.9	−270.9	−42,175.1	−46,453.7	1	III
Ten Thousand Islands NWR	137.4	−804.2	−900.2	−99,648.2	−110,907.7	2	III
Tennessee NWR	204.2	49.2	294.5	8666.4	33,136.3	3	I
Tensas River NWR	282.2	1765.3	1216.9	222,426.3	152,851.3	3	I
Texas Point NWR	33.7	372.3	144.9	43,835.5	15,812.1	1	I
Thacher Island NWR	0.1	−211.6	−234.1	−38,568.2	−39,072.8	1	III
Theodore Roosevelt NWR	25.0	917.6	998.3	121,153.2	125,599.6	8	I
Three Arch Rocks NWR	0.1	11.2	10.8	3814.4	−1585.4	1	I
Tijuana Slough NWR	4.4	−429.5	−493.4	−55,743.4	−62,414.3	1	III
Tishomingo NWR	66.6	93.0	137.0	13,330.3	17,714.2	2	I
Toppenish NWR	8.1	−343.7	−159.9	−47,560.4	−11,475.1	2	III
Trempealeau NWR	28.1	−59.7	−126.6	−13,839.1	−19,577.3	2	III
Trinity River NWR	116.0	673.8	220.9	80,705.6	25,963.3	3	I
Trustom Pond NWR	3.2	−272.8	−236.0	−47,418.9	−43,201.2	1	III
Tualatin River NWR	5.6	−631.6	−601.2	−86,866.4	−91,595.8	1	III
Tule Lake NWR	152.6	217.0	127.5	30,948.5	5007.4	2	I
Turnbull NWR	85.4	−198.3	−90.0	−29,237.6	−3036.0	1	III
Two Ponds NWR	0.3	−948.7	−818.9	−126,226.9	−122,409.8	1	III
Two Rivers NWR	30.4	−497.2	−136.3	−71,971.2	−42,465.7	2	III
Tybee NWR	2.7	−474.0	−1227.9	−74,234.8	−175,095.1	1	III
Ul Bend NWR	216.1	10.5	16.6	1918.6	3986.0	2	I
Umatilla NWR	96.2	−390.9	−121.7	−53,735.2	−5279.5	2	III
Union Slough NWR	11.8	−39.2	−36.8	−8985.5	−8203.3	2	III
Upper Klamath NWR	111.2	263.4	133.4	36,186.5	−743.0	2	I
Upper Mississippi River NWFR	634.1	−54.5	−47.9	−12,907.4	−9092.7	19	III
Upper Ouachita NWR	154.5	−10.8	−24.5	1177.1	−3198.5	1	III
Valentine NWR	263.3	18.0	29.0	1835.6	3254.8	3	I
Valle De Oro NWR	2.0	−57.7	27.0	−6254.6	−4757.5	1	II
Waccamaw NWR	105.0	120.4	−574.6	2480.2	−102,364.4	2	IV
Wallkill River NWR	24.2	−225.6	−30.1	−36,612.4	−6778.1	2	III
Wapack NWR	6.7	−1.4	28.2	−2033.6	7269.3	1	II
Wapanocca NWR	22.8	−1399.6	−1547.9	−178,219.3	−200,299.3	1	III
Wapato Lake NWR	3.9	−466.6	−467.5	−58,343.0	−76,099.1	1	III
War Horse NWR	11.5	17.7	22.2	2602.1	4836.4	4	I
Washita NWR	32.8	−96.6	197.3	−14,537.2	8250.7	1	II
Wassaw NWR	41.4	342.8	−67.5	34,260.1	−23,115.0	1	IV
Watercress Darter NWR	0.1	−317.8	−413.5	−45,256.7	−58,278.1	1	III
Waubay NWR	19.1	−18.8	−13.2	−1624.6	986.8	1	III
Wertheim NWR	10.8	−236.8	−248.0	−41,593.7	−43,262.3	3	III
West Sister Island NWR	0.3	−0.1	24.3	−939.6	2938.4	1	II
Wheeler NWR	113.8	−1126.8	−1286.4	−146,835.0	−174,621.7	1	III
White Lake NWR	4.2	−4.0	−33.8	3.7	−1489.6	1	III
Wichita Mountains WR	238.8	−10.6	193.3	−1886.9	15,292.4	2	II
Willapa NWR	68.8	−115.3	−34.4	−14,067.5	−1361.5	3	III
William L. Finley NWR	23.1	−364.9	−345.4	−36,466.9	−53,662.6	1	III
Wolf Island NWR	18.7	160.5	−237.1	11,913.2	−42,889.0	2	IV
Yazoo NWR	52.8	873.6	1028.7	116,344.1	132,811.3	1	I

Abbreviations include NWR as well as Antelope Refuge (AR), Bird Refuge (BR), Conservation Area (CA), Conservation Partnership Area (CPA), Deer Refuge (DR), Elk Refuge (ER), Fish and Wildlife Refuge (FWR), National Monument (NM), National Wildlife and Fish Refuge (NWFR), Refuge for the Columbian White‐tailed Deer (RCD), Research Refuge (RR), Waterfowl Production Area (WPA), Wildlife Refuge (WR), and Wildlife Range (WRg).

## APPENDIX 2

Forage and roosting habitat availability for dabbling ducks by migration node (32 × 32 km) in the contiguous United States. Total forage (left) and roosting habitat (right) were log‐transformed after adding a small constant. Values were estimated from the National Land Cover Database (Fry et al., 2011) by Aagaard et al. (2022). All maps use Albers equal area conical projection centered on the contiguous United States.

## APPENDIX 3

Extent of severe winter weather in a cold (left; 1957) and a warm (right; 2015) year. Contours show number of days at each location in which the WSI of Schummer et al. (2010; as implemented by Aagaard et al. (2022)), which is based on temperature and snowfall, exceeded a threshold of 7.5. Differences among years are subtle but result in substantial differences in bird responses. All maps use Albers equal area conical projection centered on the contiguous United States.

## APPENDIX 4

Relative contributions (i.e., marginal value) of migration nodes to total DUD of dabbling ducks throughout the non‐breeding period in a relatively cold (left; 1957) and a relatively warm (right; 2015) year. All estimates are based on single‐node knockouts in an energetics‐based movement and foraging model (Aagaard et al., 2022). To better display the variation among nodes while reducing the influence of extreme values, positive values were log‐transformed. For negative values, the absolute value was log‐transformed, and the negative sign was then restored. Positive and negative values were each then scaled proportionately to each other for easy comparison. All maps use Albers equal area conical projection centered on the contiguous United States.

## APPENDIX 5

Relationship between forage availability and marginal value of nodes to dabbling duck survival. Each point represents a node. Points are colored based on node baseline DUD prior to node knockout (top) and latitude (bottom). The *x*‐axis of all plots is truncated because nodes with less forage had a null contribution to survival. Contribution to survival (marginal value; *y*‐axes) was determined based on effects of node removal in the energetics‐based movement model of Aagaard et al. (2022). Latitude is based on Albers equal area conical projection centered on the contiguous United States.

## APPENDIX 6

Difference in node marginal value for dabbling duck survival in a warm (*y*‐axis) versus a cold (*x*‐axis) year. Each point represents a node, colored by baseline DUD for that node in the absence of node knockouts, averaged across a warm and a cold winter. More nodes make positive contributions in a warm winter. Marginal value for survival was determined based on effects of node removal in the energetics‐based movement model of Aagaard et al. (2022). For similar plots colored by latitude and forage availability, refer to Figure 3.

## APPENDIX 7

Node marginal value for total annual DUD of dabbling ducks, for nodes containing NWRs. Color shows contributions in a relatively cold (left) and warm (right) year. Nodes that differ in the sign of their contributions between years are shown with black outlines. To better display the variation among nodes while reducing the influence of extreme values, positive values were log‐transformed. For negative values, the absolute value was log‐transformed, and the negative sign was then restored. Positive and negative values were each then scaled proportionately to each other for easy comparison. For a map of nodes that switch from negative to positive values in a warm (vs. cold) year, refer to Appendix 8. All maps use Albers equal area conical projection centered on the contiguous United States.

## APPENDIX 8

Nodes containing NWRs switching from a net negative marginal value in cold winters to a net positive marginal value in a warm winter (in red; relative to a cold winter). Each point represents a node containing one or more refuges. Plots show nodes that change in their contribution to total DUD (left) and total survival (SURV; right). For the contribution of each node in a warm and a cold winter, refer to Figure 4 and Appendix 7. All maps use Albers equal area conical projection centered on the contiguous United States.
